# In Vitro Activity of Nisin A Against Staphylococci Isolated from Periprosthetic Joint Infection

**DOI:** 10.3390/antibiotics14050515

**Published:** 2025-05-16

**Authors:** Melissa J. Karau, Christina A. Koscianski, Andrew D. Badley, Nicholas A. Bedard, John W. Zinckgraf, Robin Patel

**Affiliations:** 1 Division of Clinical Microbiology, Department of Laboratory Medicine and Pathology, Mayo Clinic, Rochester, MN 55905, USA; 2 Division of Public Health, Infectious Diseases, and Occupational Medicine, Department of Medicine, Mayo Clinic, Rochester, MN 55905, USA; 3 Department of Orthopedic Surgery, Mayo Clinic, Rochester, MN 55905, USA; 4 ImmuCell Corp, Portland, ME 04103, USA

**Keywords:** staphylococci, nisin A, periprosthetic joint infection, vancomycin, minimum inhibitory concentration, checkerboard, biofilm

## Abstract

**Background/Objectives**: Staphylococci are the most common causes of periprosthetic joint infection (PJI); new antimicrobials are needed to manage these difficult infections. Nisin A is a lantibiotic peptide derived from *Lactococcus lactis* that has antimicrobial activity against Gram-positive bacteria, including staphylococci, and is an FDA-approved preservative used in the food and dairy industry. Here, the in vitro nisin A susceptibility of PJI-associated staphylococci was assessed. **Methods**: The minimum inhibitory concentrations (MICs), minimum biofilm inhibitory concentrations (MBICs), and minimum biofilm bactericidal concentrations (MBBCs) of nisin A were measured by broth microdilution against 106 staphylococcus isolates isolated from PJI. MICs were assessed using 5 × 10^5^ CFU/mL plus nisin A. For MBICs, biofilms were grown on pegged lids for 6 h, followed by 20 h of treatment. For MBBCs, pegged lids were transferred to plates containing media only for 20 h. The results were determined as the lowest concentrations with no visual growth. Two-dimensional MICs with nisin A and vancomycin were assessed for 20 isolates. Fractional inhibitory concentrations (FICs) were calculated to determine synergistic, additive, antagonistic, or indifferent interactions. **Results**: The MIC that inhibited 90% of *S. aureus* and *S. epidermidis* was 4 µg/mL, apart from for the MRSA subset (8 µg/mL). The MBIC that inhibited 90% of isolates was 4 µg/mL. The MBBCs ranged from 4 to 256 µg/mL. When tested together, nisin A and vancomycin yielded an FIC between 1.25 and 1.5, indicative of indifference, except for one isolate each of MRSA and MSSA, for which an additive effect (FIC of 1) was observed. **Conclusions**: Nisin A showed inhibitory activity against staphylococci that cause PJI.

## 1. Introduction

Periprosthetic joint infection (PJI) is a primary concern in total joint arthroplasty (TJA) and one of the most common causes of TJA failure. Staphylococci are the most common cause; treatment of these infections can be difficult, with surgery and long-term antimicrobial therapy often being necessary [1]. Staphylococci are not only armed with defenses of their own, such as biofilm formation and evasion of and survival in human cells [2], but there has also been a global increase in antibiotic resistance [3]. Current therapies are not fully meeting the needs required to adequately treat PJI; thus, new antimicrobials are urgently needed.

Nisin A is a class I bacteriocin, a lantibiotic peptide derived from *Lactococcus lactis*, that is FDA-approved as a preservative for use in the food and dairy industry [4]. Nisin A binds to lipid II, a cell wall precursor, and has two modes of action. First, it inhibits peptidoglycan from properly forming a cell wall. Second, it inserts itself into the cell membrane and creates pores, which result in cell lysis and death [5]. Nisin A has been reported to have bactericidal activity against planktonic staphylococci and inhibit *Staphylococcus aureus* biofilm formation. The use of nisin A as a treatment strategy in PJI is unexplored. Herein, the antimicrobial planktonic and biofilm susceptibility of nisin A was determined using 106 staphylococcal isolates from PJIs.

## 2. Results

No breakpoint for nisin A susceptibility is defined. MIC, MBIC, and MBBC results are shown in Table 1. There were limited differences between MICs and MBICs, with MIC_50_ vs. MBIC_50_ and MIC_90_ vs. MBIC_90_ being within one dilution of one another. These limited differences were also observed between species and methicillin-susceptible vs. methicillin-resistant isolates. For example, the MIC_90_ and MBIC_90_ were both 4 µg/mL for all groups analyzed, apart from the subset of MRSA, for which the MIC_90_ was 8 µg/mL. The ‘other coagulase-negative staphylococci’ tested displayed a similar susceptibility pattern, except for *Staphylococcus lugdunensis*, which had a slightly higher MIC range (4–16 µg/mL).

Nisin A’s MBBCs were higher than its MBICs, with 82% of isolates having an increase of 4 or more 2-fold dilutions. Those which did not have several dilution increases tended to have higher MBIC values. The MBBC_50_ was 64 µg/mL for all groups except MSSA, for which it was 32 µg/mL. The MBBC_90_ was 256 and 64 µg/mL for MRSA and MSSA, respectively, and 128 µg/mL for both MRSE and MSSE.

Checkboard testing between nisin A and vancomycin showed FICs between 1 and 1.5. The vancomycin and nisin A alone had an MIC_90_ (range) of 2 (0.5–2) and 8 (4–16) µg/mL, respectively. Two isolates, one each of MRSA and MSSA, had MICs of 1 and 8 µg/mL for vancomycin and nisin A, respectively. The FICs of these two isolates was 1, indicating an additive effect at vancomycin and nisin A concentrations of 0.5 and 4 µg/mL, respectively. All other isolates tested had scores between 1.25 and 1.5, indicating indifferent interactions between nisin A and vancomycin.

## 3. Discussion

Nisin A has been used in the food and dairy industry as a preservative due to its broad spectrum of antimicrobial activity and its being generally recognized as safe by the FDA [4]. With the continuing increase in antimicrobial resistance, there is therapeutic interest in the possibility of combining nisin A with traditional antibiotics for PJI management. Nisin A would likely need to be delivered directly to the joint during or after surgery or used to coat orthopedic implants. These delivery methods might allow for higher concentrations at the site of PJI than systemic delivery. However, whether or not this would be a viable management strategy remains to be shown.

Like others measuring nisin A activity against staphylococci [5,6,7,8,9,10], the nisin A MICs studied here were found to be low for PJI isolates in the planktonic form. However, in the biofilm state, we and others have found variable activity of nisin A against staphylococci [5,6,7,8,11]. There were limited differences between the MICs and MBICs, with only one two-fold dilution difference observed between planktonic and biofilm testing. This suggests that nisin A has the ability to inhibit biofilm formation, which is relevant to PJI since PJI is a biofilm-associated infection. There were limited activity differences between species as well as on the basis of methicillin susceptibility. Overall, nisin A inhibitory concentrations were not affected by methicillin resistance; however, there was a two-fold dilution increase in the MIC_90_ of MRSA compared to the other subgroups tested.

While studies on nisin A and staphylococci are limited, current evidence shows the variability of planktonic and biofilm activity using varied bacterial isolates and nisin A concentrations. Jensen et al. tested nisin A against a small collection of *S. aureus* isolates and found MICs of 6.4–12.8 µg/mL; they performed planktonic time–kill curves against *S. aureus* USA 300 JE2, finding bactericidal activity at concentrations as low as 3.2 µg/mL after 4 h, with membrane depolarization and condensed and fragmented DNA [9]. In another study, *S. aureus* MICs ranged from 1.25 to 2.5 µM (~4.19–8.39 µg/mL), and in a time–kill analysis of *S. aureus* MR23, there were no detectable planktonic cells after 4 h of 4× MIC nisin A exposure, with a ~1.5 CFU/well reduction in biofilm. In comparison, vancomycin showed no biofilm reduction after 24 h [5]. Live–dead imaging confirmed these findings, as well as a dose-dependent effect on killing, as assessed by the ATP efflux out of cells and the disruption of membrane potential [5]. Angelopoulou et al. showed the inhibition of *S. aureus* biofilm production when nisin A and vancomycin were used in combination at 1× the MIC, where nisin A or vancomycin alone only prevented biofilm formation in four and six of the eight isolates tested, respectively [6]. When treating preformed biofilms with up to 8× the MIC of nisin A or vancomycin, there was no activity; vancomycin exposure resulted in more biofilm than the controls [6], a phenomena observed previously with vancomycin [12,13,14]. In another study, nisin A alone at 6× the MIC (MIC, 1.56 µg/mL) reduced *S. aureus* SA113 biofilm compared to no treatment [11].

Nisin A synergy with antibiotics has been variable; here, two of ten isolates exhibited an additive effect with vancomycin. Dosler et al. reported synergy for four of ten *S. aureus* isolates with combinations of nisin and vancomycin in a checkerboard assay [7]. With the same isolates in a time–kill assay, nisin showed dose-dependent bactericidal activity for nine of ten isolates, with over a three-log reduction within one hour; this was further enhanced with the addition of vancomycin [7]. The reason for the lack of synergy here is unknown. Many isolates in the aforementioned studies were not from highly virulent infections, such as PJI. Performing time–kill assays may provide a more accurate portrayal of interactions between nisin A and vancomycin by assessing actual CFU counts over time.

Collectively, findings reported by others and here underline the range in activity of nisin A against staphylococcal strains in both planktonic and biofilm testing. The above reports used concentrations of nisin ranging from below the MIC to several dilutions above it. Planktonic activity against *S. aureus* has been reported, but further investigation of activity against mature biofilms is needed, particularly against clinically relevant strains and on materials associated with human biofilm infections, such as titanium used in prosthetic joints.

There were several limitations to this study. There are no Clinical Laboratory Standards Institute (Berwyn, PA, USA) or European Committee on Antimicrobial Susceptibility Testing (Växjö, Sweden) breakpoints for nisin A to classify susceptible and non-susceptible isolates. While checkerboard assays were performed, such testing is ideally suited as a screen for synergy. Time–kill curves, including planktonic and biofilm assessment, would more accurately determine nisin A and vancomycin interactions. Finally, vancomycin was the only antibiotic tested; other relevant PJI antibiotics should be tested.

## 4. Materials and Methods

Antimicrobial susceptibility testing was performed by broth microdilution using CLSI methods [15,16]. Briefly, the minimum inhibitory concentrations (MICs) of nisin A (ImmuCell Corp., Portland, ME, USA) were determined for 26 methicillin-resistant *S. aureus* (MRSA), 29 methicillin-susceptible *S. aureus* (MSSA), 27 methicillin-resistant *Staphylococcus epidermidis* (MRSE), 11 methicillin-susceptible *S. epidermidis* (MSSE), 7 *S. lugdunensis*, and 6 ‘other coagulase-negative staphylococci’ (2 *Staphylococcus capitis*, 2 *Staphylococcus caprae*, 1 *Staphylococcus hominis*, and 1 *Staphylococcus warneri*) isolated from hip or knee PJI at the Mayo Clinic. Bacteria were adjusted to 0.5 McFarland turbidity in saline solution and diluted 1:100 in cation-adjusted Mueller–Hinton II broth (CaMHB) (BD, Sparks, MD, USA), and 50 µL was transferred to a 96-well Falcon round-bottom plate (Corning Inc., Durham, NC, USA) containing 50 µL of nisin A (0.5–512 µg/mL) for a final concentration of 5 × 10^5^ CFU/mL. Plates were incubated at 37 °C for 16–20 h. The lowest concentration with no visual growth was reported as the MIC.

In addition, the minimum biofilm inhibitory concentrations (MBICs) and minimum biofilm bactericidal concentrations (MBBCs) of nisin A were determined using a described assay [17,18]. Briefly, isolates were grown in tryptic soy broth to a 0.5 McFarland turbidity standard (representing 10^8^ CFU/mL). To form biofilms, 150 µL aliquots of isolate suspensions were placed into 96-well flat-bottom Nunc^TM^ flat-bottom microtiter plates (Thermo Fisher Scientific, Roskilde, Denmark) with pegged lids (Nunc^TM^ Immuno TSP Lids, Thermo Fisher Scientific, Roskilde, Denmark) and incubated for 6 h at 37 °C on an orbital shaker. After incubation, pegged lids were rinsed in a microtiter plate containing 200 µL of phosphate-buffered saline (PBS) and transferred to a microtiter plate containing nisin A (0.5–512 µg/mL) in CaMHB with a final volume of 200 µL. Biofilms were incubated for 20 h at 37 °C, and MBICs were taken from the first wells with no visible growth. To determine MBBCs, pegged lids were rinsed with PBS to remove nisin A, transferred to a growth recovery plate containing 200 µL of CaMHB (no nisin A), and incubated for 20 h at 37 °C. MBBCs were reported as the first wells with no visible growth. The corresponding concentrations required to inhibit or kill 50 and 90% of the bacteria tested (MIC_50_ and MIC_90_, MBIC_50_ and MBIC_90_, and MBBC_50_ and MBBC_90,_ respectively) were calculated for each group with more than ten isolates. There are no established susceptibility breakpoints.

A checkboard assay was performed, as previously described [19,20], using two-dimensional broth microdilution (a similar method to MICs) with nisin A and vancomycin using a subset of the isolates above, 5 each of MRSA and MSSA, 4 each of MRSE, and MSSE, and 2 of *S. lugdunesis*. Combinations of 0.06–64 µg/mL of nisin A and 0.125–8 µg/mL of vancomycin were tested. The lowest concentrations of each combination with no visible growth were reported, and the fractional inhibitory concentrations (FICs) were calculated.

$$FIC=\frac{MIC\;of\;vancomycin\;when\;in\;combination\;with\;nisin\;A}{MIC\;of\;vancomycin\;alone}+\frac{MIC\;of\;nisin\;A\;when\;in\;combination\;with\;vancomycin}{MIC\;of\;nisin\;A\;alone}$$

The FIC index was used to determine if there was synergistic (≤0.5), additive (>0.5 to ≤1), antagonistic (>4), or neutral interactions/indifference (>1 to ≤4) at various concentrations for each isolate tested.

## 5. Conclusions

In conclusion, staphylococcal PJI is difficult to treat and prevent; new approaches are needed. Nisin A, a bacteriocin used in the food and dairy industry, showed inhibitory activity against clinical staphylococci PJI isolates as well as additive effects against some isolates when used in combination with vancomycin. Nisin A could be considered for further investigation as a potential localized PJI management strategy.

## Figures and Tables

**Table 1:** Nisin A minimal inhibitory concentration (MIC), minimal biofilm inhibitory concentration (MBIC), and minimum biofilm bactericidal concentration (MBBC) values of staphylococcal isolates (number of isolates and percentage). MRSA = methicillin-resistant *Staphylococcus aureus*; MSSA = methicillin-susceptible *S. aureus*; MRSE = methicillin-resistant *Staphylococcus epidermidis*; MSSE = methicillin-susceptible *S. epidermidis*; S. lug = *Staphylococcus lugdunensis*; other = *Staphylococcus capitis*, *Staphylococcus caprae*, *Staphylococcus hominis*, and *Staphylococcus warneri*; NA = not applicable due to low number of isolates.

	**MIC (µg/mL)**										**MIC_50_**	**MIC_90_**
	≤0.5	1	2	4	8	16	32	64	128	256		
MRSA			7 (26.9)	15 (57.7)	4 (15.4)						4	8
MSSA			11 (37.9)	17 (58.6)	1 (3.5)						4	4
MRSE		4 (14.8)	17 (63)	5 (18.5)	1 (3.7)						2	4
MSSE		1 (9.1)	7 (63.6)	3 (27.3)							2	4
S. lug				2 (28.6)	3 (42.8)	2 (28.6)					NA	NA
Other		4 (66.7)	2 (33.3)								NA	NA
	**MBIC (µg/mL)**										**MBIC_50_**	**MBIC_90_**
	≤0.5	1	2	4	8	16	32	64	128	256		
MRSA		1 (3.8)	9 (34.6)	14 (53.8)	2 (7.7)						4	4
MSSA		10 (34.5)	10 (34.5)	8 (27.5)	1 (3.5)						2	4
MRSE	2 (7.4)	8 (29.7)	13 (48.1)	2 (7.4)	2 (7.4)						2	4
MSSE	1 (9.1)	5 (45.4)	4 (36.4)	1 (9.1)							2	4
S. lug				2 (28.6)	5 (74.1)						NA	NA
Other		3 (50)	2 (33.3)	1 (16.7)							NA	NA
	**MBBC (µg/mL)**										**MBBC_50_**	**MBBC_90_**
	≤0.5	1	2	4	8	16	32	64	128	256		
MRSA						1 (3.8)	6 (23.1)	10 (38.5)	5 (19.2)	4 (15.4)	64	256
MSSA				1 (3.5)		3 (10.3)	18 (62.1)	5 (17.2)	1 (3.5)	1 (3.5)	32	64
MRSE						2 (7.4)	7 (25.9)	13 (48.1)	5 (18.5)		64	128
MSSE							2 (18.2)	6 (54.5)	2 (18.2)	1 (9.1)	64	128
S. lug					2 (28.6)	2 (28.6)	2 (28.6)	1 (14.2)			NA	NA
Other						1 (16.7)		3 (50)	2 (33.3)		NA	NA

## Data Availability

Data are available upon request.

## Abbreviations

The following abbreviations are used in this manuscript:

PJI	Periprosthetic joint infection
MRSA	Methicillin-resistant *Staphylococcus aureus*
MSSA	Methicillin-susceptible *Staphylococcus aureus*
MRSE	Methicillin-resistant *Staphylococcus epidermidis*
MSSE	Methicillin-susceptible *Staphylococcus epidermidis*
CaMHB	Cation-adjusted Mueller Hinton broth
MIC	Minimum inhibitory concentration
MBBC	Minimum biofilm bactericidal concentration
PBS	Phosphate-buffered saline
FIC	Fractional inhibitory concentration
